# Supplementary material for: Preclinical Characterization of the Distribution, Catabolism, and Elimination of a Polatuzumab Vedotin-Piiq (POLIVY®) Antibody–Drug Conjugate in Sprague Dawley Rats
Source: J Clin Med. 2021 Mar 23;10(6):1323. doi: 10.3390/jcm10061323 (PMC8004598; doi:10.3390/jcm10061323)
Supplement: Supplementary file 1 [file jcm-10-01323-s001.pdf]

Supplemental Figures

A. Unlabeled polatuzumab vedotin (DAR ~3.4)

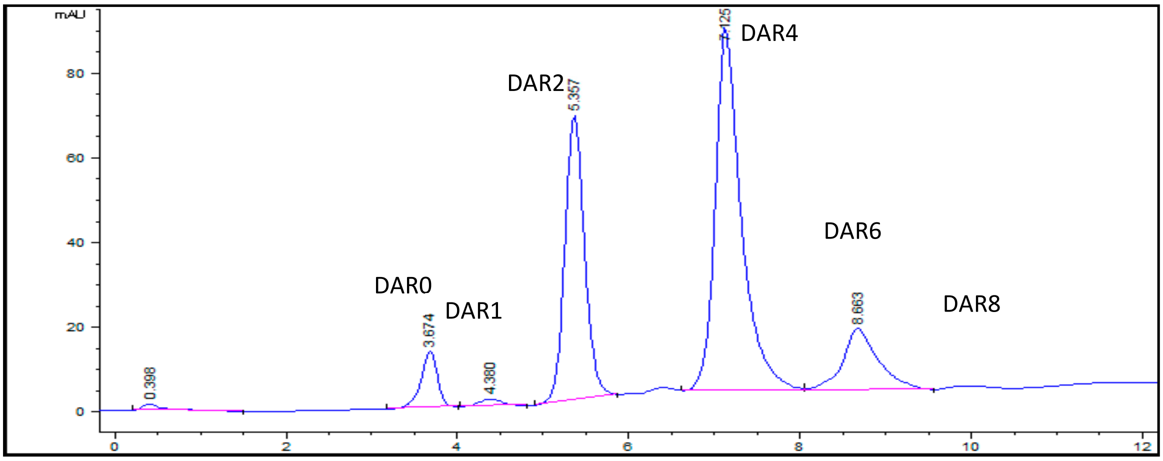

B. [^{3}H]-MMAE-polatuzumab vedotin (DAR ~3.4)

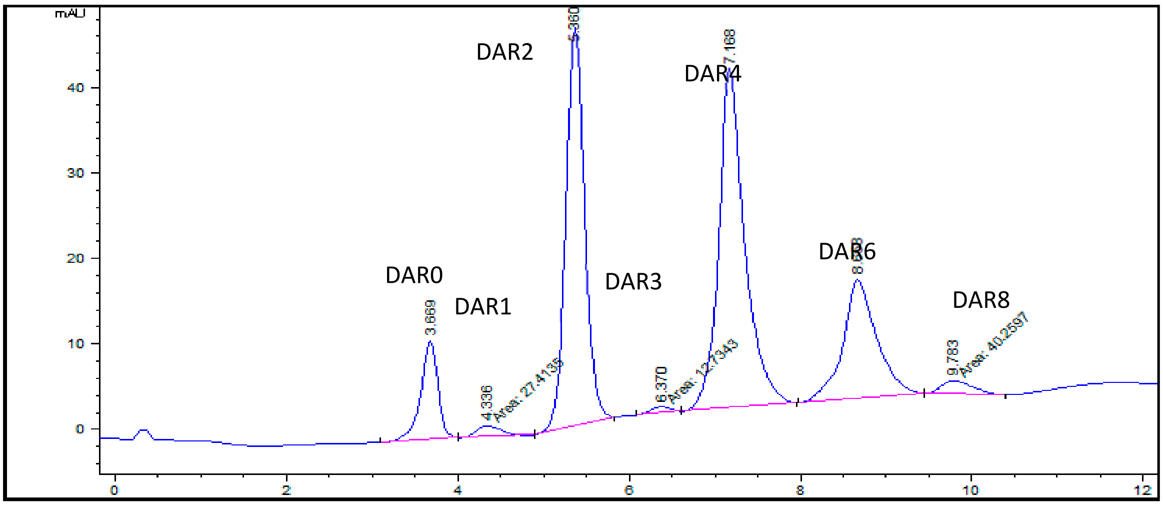

Supplemental Figure 1. The average drug to antibody ratios (DAR) were calculated from the integrated areas of the DAR species resolved by hydrophobic interaction chromatography detected at UV 280 nm. (A) Unlabeled reference polatuzumab vedotin with a DAR ~3.4. (B) [^{3}H]-MMAE-polatuzumab vedotin with a DAR ~3.4.

A.

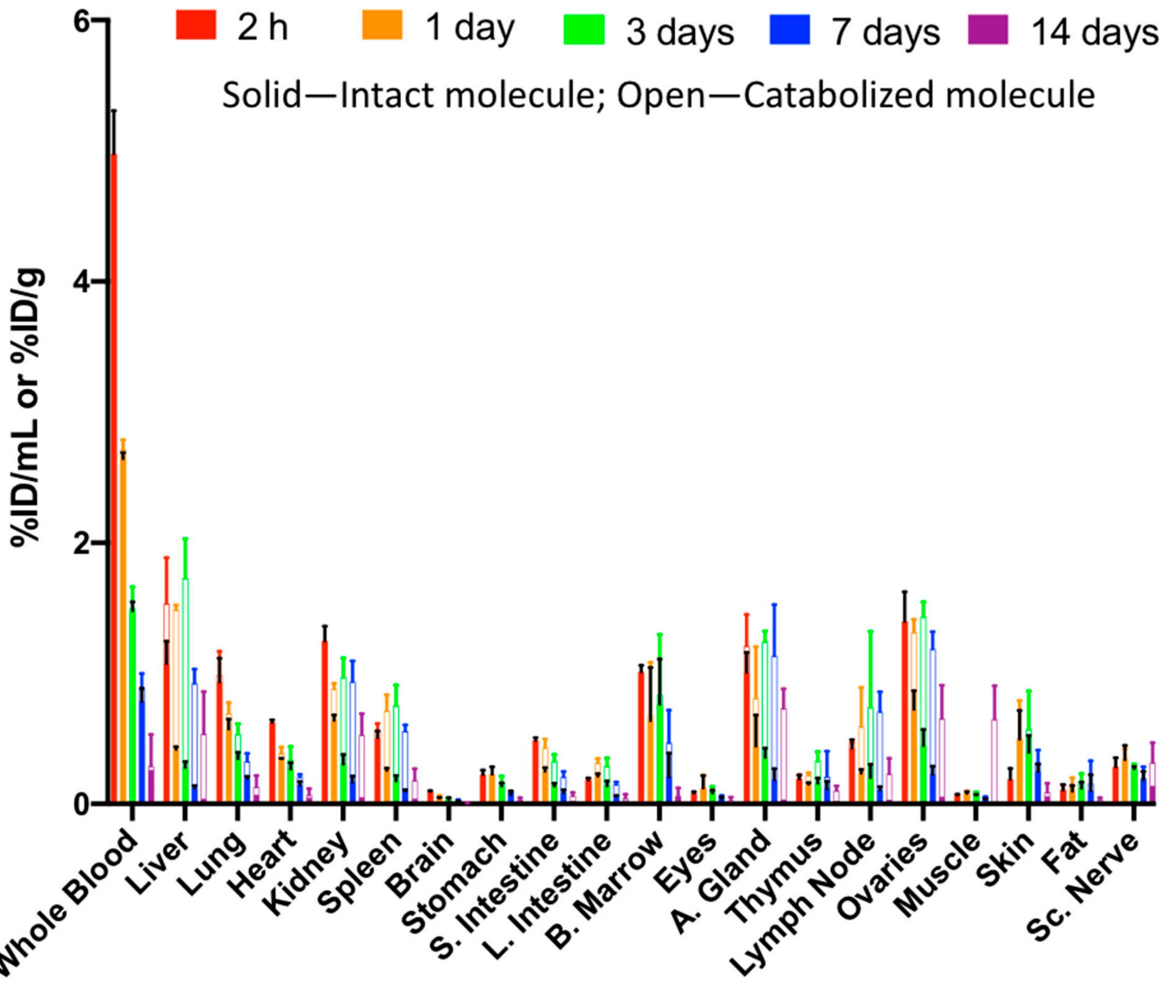

2 h
1 day
3 days
7 days
14 days
Solid—Intact molecule; Open—Catabolized molecule
%ID/mL or %ID/g
6
4
2
0
Whole Blood
Liver
Lung
Heart
Kidney
Spleen
Brain
Stomach
S. Intestine
L. Intestine
B. Marrow
Eyes
A. Gland
Thymus
Lymph Node
Ovaries
Muscle
Skin
Fat
Sc. Nerve

B.

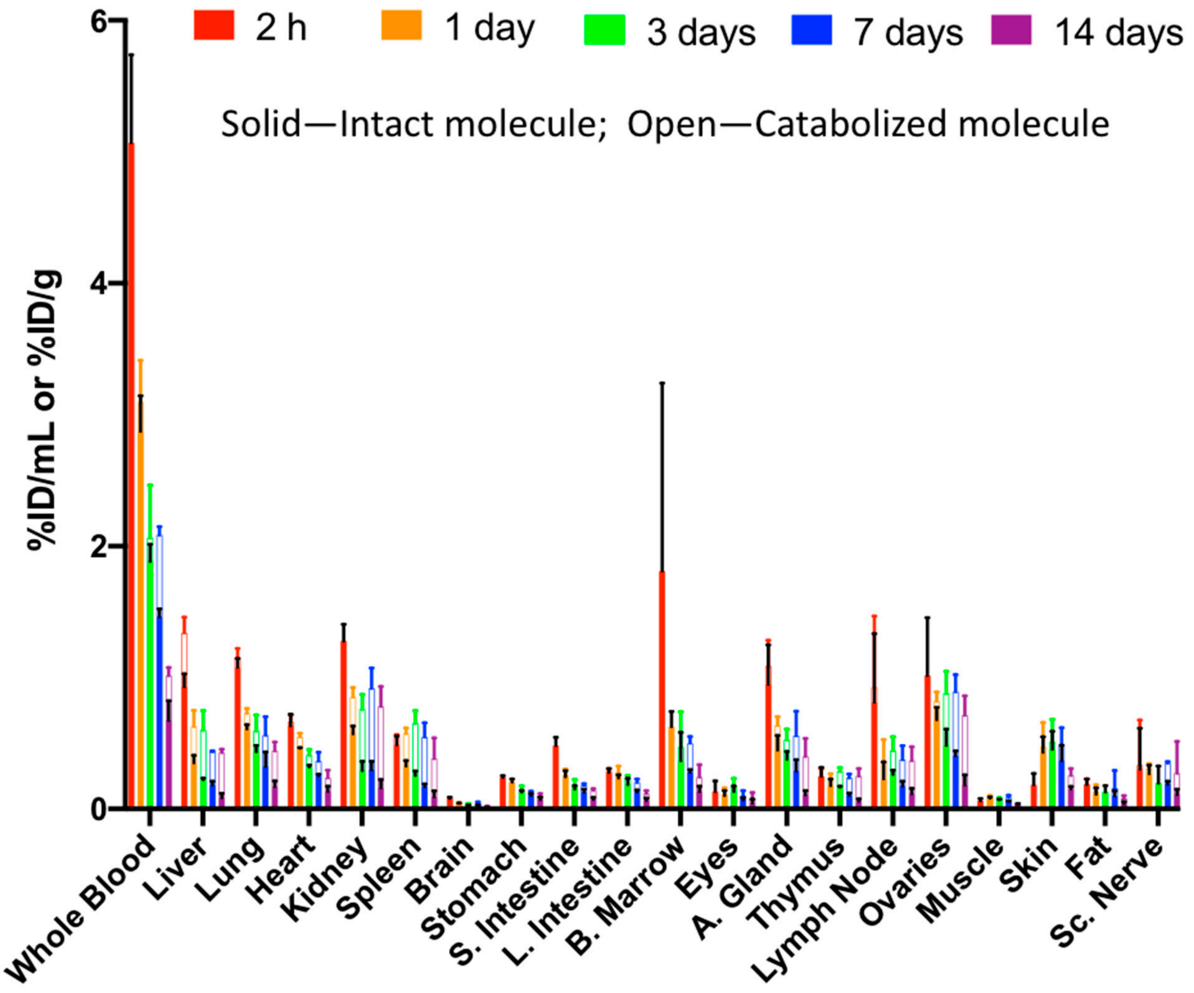

Supplemental Figure 2. The tissue distribution of polatuzumab vedotin and polatuzumab antibody after a single dose via IV administration. (A) The tissue and blood radioactivity from intact (^{125}I radioactivity—solid bar) and catabolized (^{111}In subtracted with ^{125}I radioactivity—open bar) polatuzumab vedotin dosed at tracer (~3–4 ug/kg) plus 10 mg/kg of unlabeled polatuzumab vedotin up to 14 days post administration. (B) The tissue and blood radioactivity from intact (^{125}I radioactivity—solid bar) and catabolized (^{111}In subtracted with ^{125}I radioactivity—open bar) polatuzumab antibody dosed at tracer (~3–4 ug/kg) plus 10 mg/kg of

unlabeled polatuzumab antibody up to 14 days post administration. The data are represented as the mean ± SD. n = 3 per time point. S. Intestine: small intestine; L. Intestine: large intestine; B. Marrows: bone marrows; A. gland: adrenal glands; DRG: dorsal root ganglions.
